# Debunking a myth: plant consciousness

**DOI:** 10.1007/s00709-020-01579-w

**Published:** 2020-11-16

**Authors:** Jon Mallatt, Michael R. Blatt, Andreas Draguhn, David G. Robinson, Lincoln Taiz

**Affiliations:** 1 The University of Washington WWAMI Medical Education Program at The University of Idaho, Moscow, ID 83844 USA; 2 Laboratory of Plant Physiology and Biophysics, Bower Building, University of Glasgow, Glasgow, G12 8QQ UK; 3 Institute for Physiology and Pathophysiology, Medical Faculty, University of Heidelberg, 69120 Heidelberg, Germany; 4 Centre for Organismal Studies, University of Heidelberg, 69120 Heidelberg, Germany; 5 Department of Molecular, Cellular, and Developmental Biology, University of California, Santa Cruz, CA 95064 USA

**Keywords:** Plant and animal consciousness, Plant electrophysiology, Proactive behavior, Reciprocal signaling, Classical (Pavlovian) learning, Cell consciousness

## Abstract

Claims that plants have conscious experiences have increased in recent years and have received wide coverage, from the popular media to scientific journals. Such claims are misleading and have the potential to misdirect funding and governmental policy decisions. After defining basic, primary consciousness, we provide new arguments against 12 core claims made by the proponents of plant consciousness. Three important new conclusions of our study are (1) plants have not been shown to perform the proactive, anticipatory behaviors associated with consciousness, but only to sense and follow stimulus trails reactively; (2) electrophysiological signaling in plants serves immediate physiological functions rather than integrative-information processing as in nervous systems of animals, giving no indication of plant consciousness; (3) the controversial claim of classical Pavlovian learning in plants, even if correct, is irrelevant because this type of learning does not require consciousness. Finally, we present our own hypothesis, based on two logical assumptions, concerning which organisms possess consciousness. Our first assumption is that affective (emotional) consciousness is marked by an advanced capacity for operant learning about rewards and punishments. Our second assumption is that image-based conscious experience is marked by demonstrably mapped representations of the external environment within the body. Certain animals fit both of these criteria, but plants fit neither. We conclude that claims for plant consciousness are highly speculative and lack sound scientific support.

## Introduction

The idea that plants are conscious is increasingly promoted by a vocal handful of botanists (A. Nagel 1997; Calvo 2017; Calvo et al. 2017; Gagliano 2017, 2018; Calvo and Trewavas 2020; Trewavas et al. 2020). This continues despite rebuttals of the claim by mainstream plant biologists (Alpi et al. 2007; Robinson et al. 2018; Taiz et al. 2019, 2020), and the idea has received widespread coverage in the popular press and media (https://www.newyorker.com/magazine/2013/12/23/the-intelligent-plant; https://e360.yale.edu/features/are_trees_sentient_peter_wohlleben; https://www.youtube.com/watch?v=Xm5i53eiMkU; https://www.wnycstudios.org/podcasts/radiolab/articles/smarty-plants). Proponents of plant consciousness also champion the concepts of “plant neurobiology” and “plant intelligence” (Brenner et al. 2006). Plants do not have neurons, but plant-neurobiology proponents claim they have analogous structures. In many cases, these proponents treat plant intelligence and plant consciousness with little distinction, using the same arguments for both attributes (Trewavas and Baluska 2011; Leopold 2014; Trewavas 2016; Reber and Baluška 2020; Trewavas et al. 2020).

Here, we disentangle the “intelligence” concept (Chamovitz 2018) from the “consciousness” concept to focus on the claims that are explicitly concerned with plant consciousness. We list 12 such claims (Table 1) and analyze them individually. We then present an alternate hypothesis of which organisms have consciousness, a hypothesis that fits the widespread scientific view that consciousness is an emergent property arising from complex networks of neurons (Feinberg and Mallatt 2020). We provide many new arguments against plant consciousness, plus new angles on past arguments.

**Table 1:** Claims for plant consciousness

1. Each living cell is conscious.	
2. Consciousness in plants is indicated because they sense environmental changes and respond adaptively, integrating information for goal-directed behaviors, and making decisions along the way.	
3. Membrane potentials and electrical signals are similar in plants and animals, in ways that allow consciousness.	
4. Action potentials and other electrical signals for communication propagate, neuron-like, along phloem elements.	
5. Plants, like animals with neurons, use electrical signals to integrate information for consciousness.	
6. Plants have a brain (“command center”) in the root.	
7. Plants show proactive, anticipatory behavior.	
8. Plants show classical associative learning, which indicates consciousness.	
9. Plants communicate with each other in a purposeful manner and, hence, have conscious self-recognition.	
10. Detailed hypotheses, predictions and models can substitute for hard evidence of plant consciousness.	
11. Plants show affective (emotional) consciousness.	
12. Plants have image-based consciousness, based on internal representations.	

## Definition of consciousness

Consciousness is a difficult topic, and its constructs and definition are much debated (https://en.wikipedia.org/wiki/Category:Consciousness#:). Even so, considerable agreement is achieved when it is stripped to its fundamentals. Both we and the proponents of plant consciousness focus on the most basic type, called phenomenal or primary consciousness (Block 1995; Edelman et al. 2011; Feinberg and Mallatt 2016a, b; Calvo 2017; Mallatt and Feinberg 2020). Primary consciousness means having *any* type of experiences or feelings, no matter how faint or fleeting (Revonsuo 2006: p. 37). Such a basal type of consciousness was most succinctly characterized by Thomas Nagel (1974) as “something it is like to be” when he asked, “What is it like to be a bat?” It means having a subjective or first-person point of view, and what is sometimes called sentience (from Latin *sententia*, “feeling”). This primary form of consciousness does *not* involve the ability to reflect on the experiences, the self-awareness that one is conscious, self-recognition in a mirror, episodic memory (the recollection of past personal experiences that occurred at a particular time and place), dreaming, or higher cognitive thought, all of which are higher types of consciousness (Feinberg and Mallatt 2018: p. 131). All conscious organisms have primary consciousness, but only some of them have evolved higher consciousness on that base.

Restricting our discussion to primary consciousness lets us focus on the minimal criteria for consciousness in plants. There is already abundant evidence that consciousness in animals depends on the presence of a brain and nervous system. However, many proponents of plant consciousness have argued that plants need not have human-type or animal-type consciousness (Trewavas et al. 2020). Instead, they propose that plants have something more “alien” that is nonneural yet still fits the criterion for primary consciousness of raw experience—that is, something it is like to be (Calvo 2017).

There is more to the definition of primary consciousness than indicated thus far. First, the raw experience of primary consciousness is divided into two types or aspects (Feinberg and Mallatt 2016a):

1. Experiencing a mental image or representation of the sensed world.
2. Experiencing affective feelings. Affective essentially means emotional consciousness, which in its simplest form is feelings of good or bad.

Second, primary consciousness is also “understood as the capacities to be aware of the environment and to integrate sensory information for purposeful organismal behavior.” This statement came from a plant-consciousness paper (Trewavas et al. 2020) and it is a proper characterization (Feinberg and Mallatt 2018), *but only if* (1) “aware” has its true, dictionary definition as a felt sensory experience and is not misconstrued as mere sensory reception; and (2) “purposeful” means “volitional,” rather than merely “adaptive” in the evolutionary sense of being programmed by natural selection.

## Claims for plant consciousness

### Claim 1: each living cell is conscious

Proponents of plant consciousness make two contradictory claims: (1) that consciousness emerged in the first cells *before the evolution of plants* (Trewavas and Baluska 2011; Baluska and Reber 2019; Calvo et al. 2020), and (2) that it evolved *with the first plants* (Calvo 2017; Trewavas 2017). The proponents even made both claims in the *same* paper (Trewavas et al. 2020). So, which is it? This contradiction needs to be explicitly resolved because plant consciousness cannot have any special meaning if it is nothing more than cell consciousness.

Proponents of plant consciousness use a theory called the Cellular Basis of Consciousness or CBC (Reber 2016). Here, we enumerate the objections to this theory, new and old (Key 2016; Mallatt and Feinberg 2017). First, the proponents of CBC seem to equate the fact that cells have sensory receptor molecules (and sensory-response actions) with having *conscious* sensory perception. They also assume that cellular actions must either be hard-wired (robotic: Reber and Baluška 2020) or else conscious, without appreciating the large amount of adaptive plasticity in cell physiology that can produce complex actions without any consciousness. Additionally, they do not realize that the simple forms of learning of which cells are capable—only nonassociative learning (van Duijn 2017)—do not reflect consciousness, according to behavioral scientists (see Claim 8 below).

Another objection to the idea of cell consciousness is that it traps its proponents in an absurd conclusion about consciousness in *humans*, the organisms that are most verifiably conscious. After stating that consciousness arose in the first, prokaryotic, cells, Baluska and Reber (2019) write, “whatever mechanisms [for sentience] operate at the level of prokaryotes will carry on their functions in eukaryotes and multicellular organisms [because] a basic principle of evolutionary biology is that adaptive forms and functions, once established are rarely jettisoned…” Apart from the fact that their “basic principle” is incorrect because evolutionary loss of traits is common (Bleidorn 2007; Ellers et al. 2012; Futuyma and Kirkpatrick 2017), the claim that all cells are conscious necessarily means that all human cells are conscious, never having lost their prokaryote-based consciousness. However, the fact that only brain injuries diminish human consciousness, whereas the loss of our somatic cells does not, is evidence against this idea (also see Ginsburg and Jablonka 2020, 2021).

Arthur Reber addressed the thorny problem of how the separate consciousnesses of our trillions of cells could fit with our single, unified, brain-based consciousness. He proposed that the body’s many cells, through extensive intercellular communication, “turned over” (some of?) their consciousness to the nervous system when the latter evolved, while still retaining their individual cellular consciousnesses (Reber 2019, pp. 195-196; Reber and Baluška 2020, p. 3). It is probably untestable, however, and Reber admitted his was a “speculative framework” without supporting evidence.

The idea of cell consciousness also strains credulity with its claim about the origin of *plant* cellular consciousness. Baluska and his colleagues have proposed that a eukaryotic plant cell is a supracellular unit derived from four ancestral prokaryotic cells (1. cytoplasm and plasma membrane; 2. nucleus; 3. chloroplasts; and 4. mitochondria), whose four consciousnesses became integrated into a single consciousness during evolution (Baluška and Mancuso 2014; Baluška and Miller 2018; Baluška and Reber 2019). Such an extraordinary claim about consciousness requires a substantial amount of hard evidence, but no such evidence was provided.

A major argument used by proponents of cell (and plant) consciousness is that some unicellular organisms can travel over distances in a directed manner, even navigating mazes, to reach a target as if by anticipatory, proactive behavior (Trewavas 2017; Baluska and Reber 2019). Recently, Tweedy et al. (2020) delivered a blow to this interpretation. These investigators studied social amoebas and human cancer cells that followed stimulus trails by chemotaxis as the cells detected attractant molecules with receptors on their cell membranes. The migrating cells removed and broke down attractant molecules that had diffused toward them, then they detected and followed the resulting, altered attractant gradient. The key to this was that the cells lowered the concentration of attractant *ahead of them*. Through this process, the cells readily navigated to the attractant’s source, even following the gradients *ahead around corners and through mazes*. Therefore, such migratory tracking, which proponents of cell consciousness claim requires intelligent, anticipatory consciousness, is instead fully explainable by a simple mechanism of reception-breakdown-response, with no need to invoke cellular consciousness.

### Claim 2: consciousness in plants is indicated because they sense environmental changes and respond adaptively, integrating information for goal-directed behaviors and making decisions along the way

This claim comes from an article by Trewavas et al. (2020). The term “goal-directed behavior” was defined by evolutionary biologist Ernst Mayr (2004: pp. 51-53) to mean going toward an adaptive endpoint via an evolved, usually genetic, program. By this definition, “goal-directed behavior” applies not only to consciousness but to nonconscious, physiological processes as well. All living organisms perform the adaptive, physiological behaviors of receiving, processing, and responding to stimuli—and we have argued above that not all life is conscious. Therefore, the fact that plants have these behaviors does not make them conscious (Ginsburg and Jablonka 2021; Hamilton and McBrayer 2020).

### Claim 3: membrane potentials and electrical signals are similar in plants and animals, in ways that allow consciousness

Plant cells have membrane potentials and they propagate potential fluctuations that can induce events elsewhere within the plant’s body, either nearby or at a distance (Fromm and Lautner 2007; van Bel et al. 2014; Gallé et al. 2015; Zimmermann et al. 2016; Klejchova et al. 2021). But how similar are these to the electrical signals carried by animal neurons? Proponents of plant consciousness claim a strong homology:

The working hypothesis of plant neurobiology is that the integration and transmission of information at the plant level involves neuron-like processes (Calvo and Trewavas 2020: p. 1).

Animal-plant similarities being reported in the last decade point toward an electrochemical equivalency at the level of the nervous system elements . . . (Calvo et al. 2017: p. 2866, after Baluška 2010).

The problem with these statements is that there is no “electrochemical equivalency” between animals and plants. Electrical activity in plants is powered by transport of H^+^ and that of animals by transport of Na^+^ (Canales et al. 2018). Moreover, the electrical and chemical components of the electrochemical gradient as defined in the Nernst-Planck equation are different: in plants, typically 50–70% of the free energy generated by the plasmalemma H^+^-ATPases goes into the electrical component (the membrane voltage), the rest into the pH gradient. In animals, it is the other way around: roughly 80–90% of the electrochemical gradient generated by the Na^+^/K^+^-ATPases goes into the chemical gradient of Na^+^ (and K^+^), and only a small fraction goes into the voltage difference across the membrane (Alberts et al. 2014; Klejchova et al. 2021).

Proponents of plant consciousness also latch onto the fact that all cells regulate ion fluxes across their plasmalemma to survive and then they conflate this universal property of life with “electrical signaling” to imply consciousness. They ignore the fact that regulated ion fluxes and propagating electrical signals (such as action potentials) exist in many nonneuronal tissues, including those of animals, without any role in processing or integrating information. In short, the presence of electrical activity is not a useful criterion for identifying consciousness in plants.

The following list of signal differences applies to the tracheophyte land plants that are the focus of almost all the literature on “plant consciousness.”

i. Plant cells lack the rapidly activating, voltage-dependent Na^+^ channels that give rise to action potentials in animals (Edel et al. 2017). Instead, plant action potentials (AP) are normally initiated by Ca^2+^ influx (from both external and internal sources), followed by depolarizing Cl^−^ and repolarizing K^+^ fluxes. The resulting all-or-nothing AP is not merely a propagating voltage signal, but also travels together with, and at the same speed as, a concomitant rise in cytosolic Ca^2+^ (Chen et al. 2012; Minguet-Parramona et al. 2016; Klejchova et al. 2021). Such propagating calcium elevations also occur in animals for nonneural functions, for example during contraction of the smooth musculature of blood vessels in vertebrates (Borysova et al. 2018). Indeed, the Ca^2+^ waves in plants have multiple, direct physiological functions that are unlike those of animal neurons. They coordinate local solute fluxes to adjust turgor of plant cells (Minguet-Parramona et al. 2016), they signal the presence of pathogenic infections, and they affect mass flow through the vasculature (van Bel et al. 2011, 2014; Klejchova et al. 2021). Thus, having action potentials linked to slow calcium elevations gives no indication of neuron-like information processing.
ii. Plant action potentials travel more slowly than those of animals, 0.04–0.6 m s^−1^ versus 0.5–100 m s^−1^, respectively, and with long refractory periods between successive action potentials (Fromm and Lautner 2007; Canales et al. 2018). Only in specialized organs, like the Venus flytrap, are refractory periods shorter, for faster action (Scherzer et al. 2019).
iii. Plant action potentials cause a net outflow of K^+^ and Cl^−^ ions, whereas animal action potentials are osmotically neutral, suggesting that plant action potentials function in osmotic regulation (Taiz et al. 2019; Klejchova et al. 2021). The role of action potentials in osmotic adjustment is demonstrably the case in stomatal guard cells (Chen et al. 2012; Jezek and Blatt 2017). The idea that plant action potentials have their origins in osmoregulation rather than communication is also consistent with the fact that they occur in green algae, the sister group of land plants (Köhler et al. 1983; Thiel et al. 1997).
iv. Electrical-potential fluctuations in plants are highly diverse, based on many different subtypes of ion channels and pumps. These signals also differ according to their location in the plant (root, stem, shoot, etc.), their stage in the life cycle, and their taxonomic group (Zimmermann et al. 2016; Canales et al. 2018: p. 10179; Nguyen et al. 2018). This variety is analogous to the diverse actions of membrane- and transepithelial potentials in different organs of animals (Bartos et al. 2015; Kadir et al. 2018), but not to the specialized, fast potential fluctuations in nervous tissue. The nervous signals are much more uniform, being constrained for optimal speed, energy efficacy, and information transfer.
v. Glutamate and its receptors are important for neurotransmission in animals, and glutamate receptors exist in plants. However, their major role in plants appears to be mediating Ca^2+^ flux, rather than acting in neurotransmission (Forde and Roberts 2014; Nguyen et al. 2018; Taiz et al. 2020; Klejchova et al. 2021).

Finally, it must be stressed that electrical signaling in plants is far less understood than in animals, which should caution researchers against speculating beyond the evidence to assign similarities.

### Claim 4: action potentials and other electrical signals for communication propagate, neuron-like, along phloem elements (Calvo et al. 2017)

**Fig. 1:**
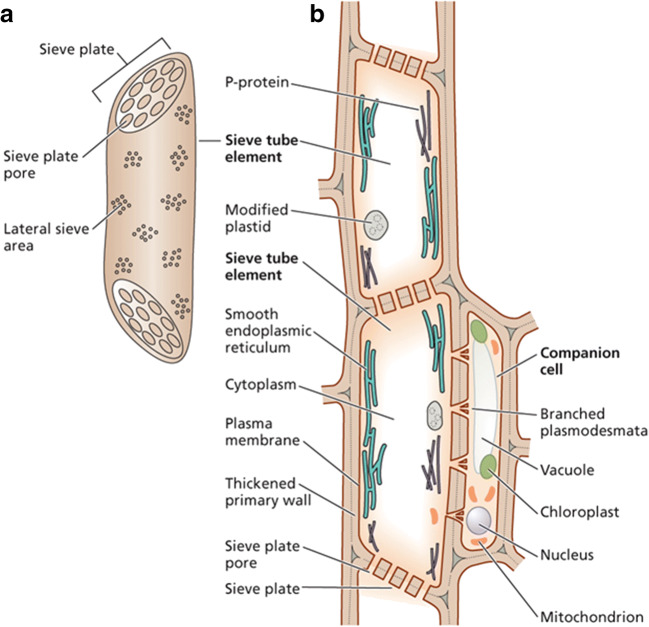
Phloem vascular system. **a** Sieve element. **b** Sieve tube consisting of seive elements. Reprinted with permission from Taiz et al. (2015), Sinauer, Oxford University Press

The phloem of the vascular system carries electrical signals for considerable distances within plants (Fromm and Lautner 2007; Canales et al. 2018). Phloem consists of electrically excitable cells called *sieve elements*, connected to one another in a column (*sieve tube*) at junctions called *sieve plates* (Fig. 1). However, signal transmission along the phloem differs in notable ways from that on neuronal axons.

The *action potentials* carried by phloem differ from animal action potentials, as noted above, by encompassing an osmoregulatory function. Phloem APs also signal distant changes in cellular photosynthesis, respiration, and phloem transport (Fromm and Lautner 2007; Fromm et al. 2013), but these are arguably caused by a primarily osmotic mechanism: in every case where plant action potentials are documented with mechanistic precision in a physiological response, the central mechanism is osmotic (guard cells and photosynthetic gas exchange, characean algae, carnivorous plants, leaf movements, etc.: Beilby 2007; Jezek and Blatt 2017; Lawson and Matthews 2020).

Phloem-conducted action potentials in plants are commonly responses to noninvasive and nondamaging environmental stimuli, such as touch, cooling, and light (Fromm and Lautner 2007; Fromm et al. 2013; van Bel et al. 2014; Gallé et al. 2015). By contrast, the defense responses to destructive wounding and burning injuries are signaled by *variation potentials* (VPs, sometimes called “slow wave potentials”), which are also conducted by the phloem. Accompanying these VPs are waves of Ca^2+^ and reactive oxygen species in the cytoplasm (van Bel et al. 2014; Evans and Morris 2017; Nguyen et al. 2018; Toyota et al. 2018; Lew et al. 2020; Klejchova et al. 2021), with the defense responses including accumulation of jasmonate, salicylic acid, ethylene, and other adaptations to stress.

VPs merit special attention because they are *especially relevant to the question of whether plants have the conscious experience of pain.* That is, injury-induced VPs are the closest functional analogues in plants to the nociceptive neural signals that lead to conscious pain in animals. Nociception in animals is the nonconscious sensing of injurious stimuli and is not itself pain, but it is processed into pain by higher-level neuronal signaling (Draguhn et al. 2020). Therefore, if the electrical properties of plant VPs resemble nociceptive signals, then it is conceivable that plants could also feel pain. Does such a resemblance exist?

No, VPs are different from nociceptive action potentials, and from anything expected to code for consciousness. Plant VPs travel slowly, at only about 0.001 m s^−1^ (Zimmermann et al. 2009; Mousavi et al. 2013), far below the 0.5–2 m s^−1^ of the *slow nociceptive* action potentials that propagate along human axons after wounding (Purves et al. 2018). Unlike action potentials, new VPs can be generated only every 10 min to several hours (Klejchova et al. 2021) and they decay over time and distance (decreasing in amplitude). Each VP is unitary and long-lasting (for over 5 min: Nguyen et al. 2018). VPs cannot signal all the way from one end of a plant to another, either by amplitude or velocity. Another characteristic that precludes neuron-like encoding by VPs is that they are highly variable in amplitude and temporal behavior, unlike the frequency encoding that characterizes electrical spike trains in neurons and is necessary for consciousness in animals (Dennett 2015; Klejchova et al. 2021).

In summary, phloem transmits APs and VPs, neither of which is similar to signal transmission in neural axons. The VPs seem especially unsuitable for any role in consciousness.

### Claim 5: plants, like animals with neurons, use electrical signals to integrate information for consciousness

*Information integration* has a detailed, formal definition (Tononi and Koch 2015; Koch 2019) that roughly means the parts of a system interact so the outputs differ from the mere sum of the inputs. For plant neurobiology, however, Calvo (2017: p. 212) treats information integration as combining and processing diverse information to make decisions about responses. That is the sense in which we use the term here.

**Fig. 2:**
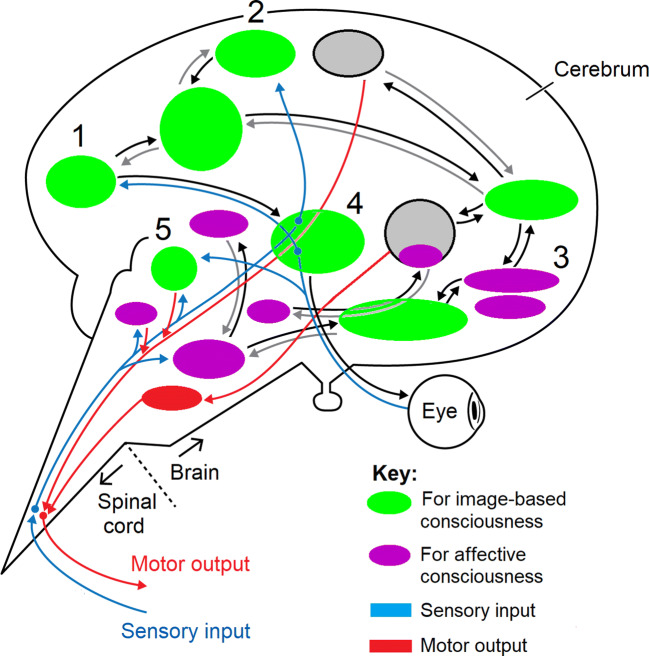
Extensive reciprocal communication (back-and-forth arrows) between processing centers (ovals) in the human brain is an indicator of consciousness. Such integrative communication also occurs *within* the centers (not shown). This is a side view of the brain, with anterior to the right. For simplicity, we only label/number a few of the main centers: 1. primary visual cortex of the cerebrum; 2. somatosensory cortex; 3. amygdala (for fear and other emotions); 4. thalamus; 5. superior colliculus of midbrain (optic tectum). Modified from Fig. 3 in Feinberg and Mallatt (2020)

The consensus of opinion among those who study consciousness in animals is that it depends on information integration that involves extensive feedback, or reciprocal (recurrent) communication, between the conductive neurons (Lamme 2006; Feinberg and Mallatt 2018; Koch 2019; Mashour et al. 2020). This reciprocal connectivity is shown in a human brain in Fig. 2. Such integrative electrical signaling is easily recorded among neurons in the brains of conscious humans as well as in brains of other mammals performing the same mental tasks (Fahrenfort et al. 2007; Storm et al. 2017), *but it has never been detected in plant phloem or any other part of a plant. That is, forward signals are documented but feedback signals have not been found.* Such integrating signals have merely been hypothesized to occur (Calvo et al. 2017: p. 2866; Taiz et al. 2020). In legumes, a type of reciprocal signaling occurs between shoots and roots to regulate the formation of root nodules that contain N_2_-fixing bacteria, but this is different: only the first, local signaling steps are electrical whereas most of the steps are nonelectrical and involve long-distance movements of peptides via the vascular system (Krusell et al. 2002; Damiani et al. 2016; Roy et al. 2020).

**Fig. 3:**
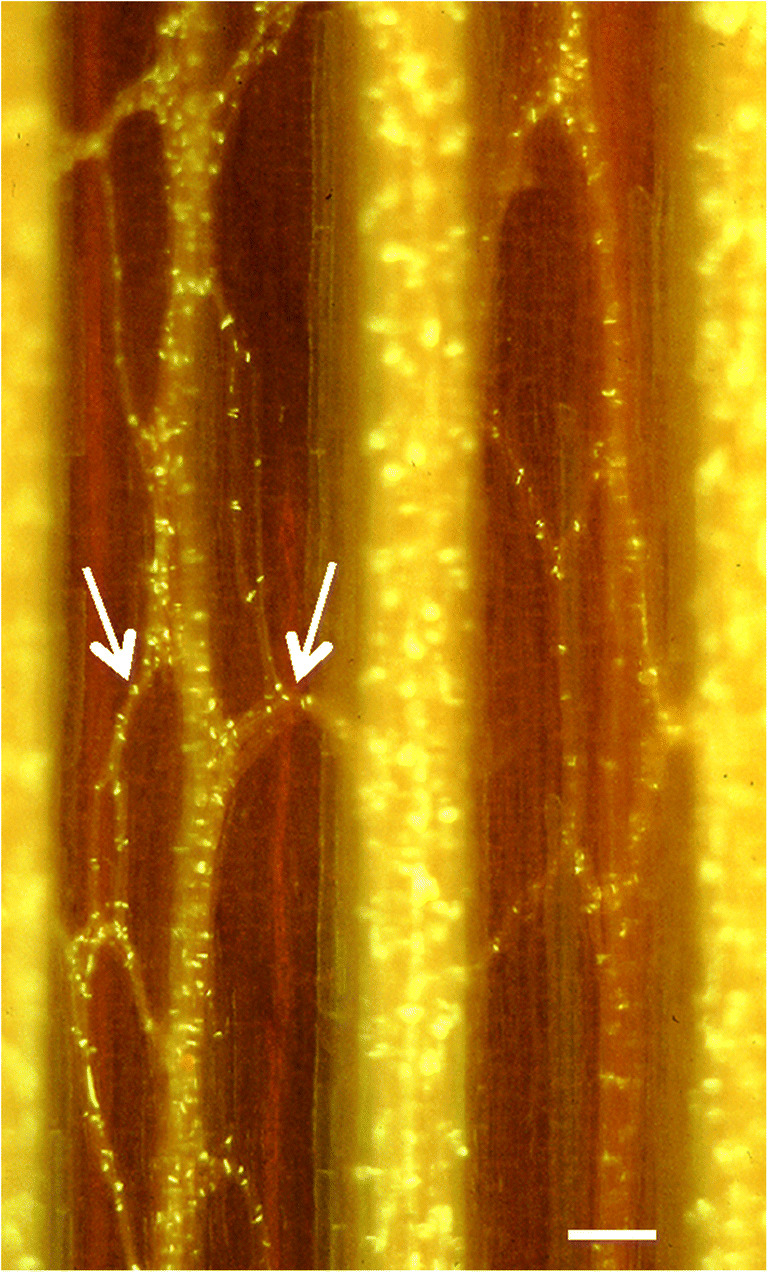
Longitudinal view of the phloem in *Dahlia pinnata*. Patterns of phloem anastomoses (arrows) are evident between the longitudinal vascular bundles. The phloem was removed from the xylem at the cambial zone, and is shown from the cambium side in an intact stem, stained with aniline blue and observed under epifluorescence microscope. Scale bar = 100 μm. Micrograph is a gift from Roni Aloni

For the information integration of consciousness, another generally accepted requirement is a high degree of interconnectivity among neurons. An average neuron in the human brain contacts about 10,000 other neurons (Zhang 2019), through its many branching processes and synapses. In contrast, the phloem vascular bundles in the internodes of plants are primarily unbranched and linear, and both sugar translocation and signaling occur along this linear axis. However, branches with anastomoses (interconnections) can occur between adjacent bundles to form a network for the lateral movement of water, minerals, and photosynthetic products (Fig. 3). However, these anastomoses are not more elaborate than in blood-vessel networks of animals (Kopylova et al. 2017; Alves et al. 2018), which are not involved with consciousness. Even so, they led Calvo et al. (2017) to suggest that phloem networks have the additional function of generating consciousness in plants, by analogy to the neuronal networks in animal brains.

A difficulty with this argument is that phloem anatomoses are absent in *young* internodes (Aloni and Barnett 1996). If phloem anatomoses are required for plant consciousness, the growing tip and younger internodes of a plant would be nonconscious, even though these are essential for regulating tropistic curvatures in growing plants and nutational movements—actions that are often cited as outward expressions of plant consciousness (Gagliano et al. 2016; Calvo 2017: p. 219; Calvo and Trewavas 2020). Phloem anastomoses are also absent in young germinating seedlings, yet seedlings still exhibit many of the behaviors of mature plants. If the function of plant consciousness is to allow plants to make important “decisions,” why would it be active in mature plants but not in young seedlings, the most vulnerable part of the life cycle?

For other arguments against phloem anastomoses resembling neuronal networks, see Taiz et al. (2020).

### Claim 6: plants have a brain (“command center”) in the root

From early comments by Charles Darwin (1880) on the ability of the root tip to control the direction in which a root grows, Baluska and colleagues consider this tip a “brain-like command center” (Baluška et al. 2004, 2009; and the “somatic mosaic” idea in Calvo et al. 2020). Baluška and Hlavačka (2005) and Baluška et al. (2009) cite actin-rich domains in these root cells as evidence of endocytosis and vesicle recycling reminiscent of neuronal synapses. However, there is no clear cytological evidence for synapses in plants (Hertel 2018; Robinson et al. 2018; Taiz et al. 2019, 2020).

**Fig. 4:**
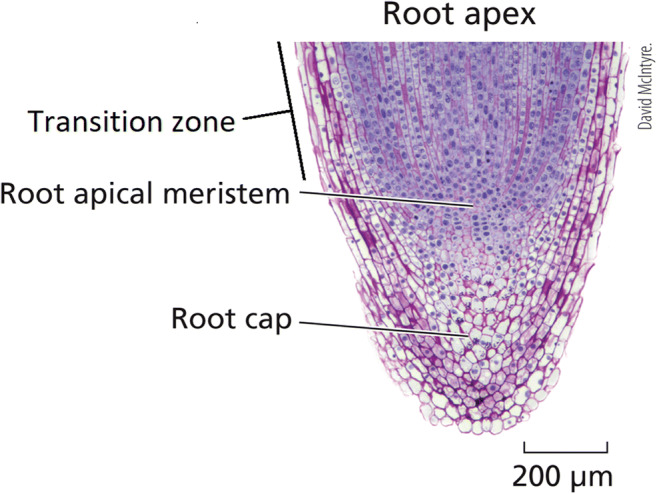
Histomicrograph of a root tip, from flax (*Linum usitatissimum*). The *zone of elongation* lies just above the top of the micrograph. Reprinted with permission from Taiz et al. (2015), Sinauer, Oxford University Press

Moreover, the transition zone of the root tip (Fig. 4), between the apical meristem and the zone of elongation, is a peculiar place to situate a putative brain-like organ for consciousness and memory storage. In the first place, the dividing cells of this transition zone are immature and undifferentiated (Salvi et al. 2020), unlike functioning neurons, which are mature and fully differentiated. By analogy, the dividing, undifferentiated pre-neurons in the embryonic vertebrate brain have not yet developed their cell processes or formed functioning networks required to generate consciousness (Sadler 2018).

A second objection to the “transition zone equals brain” proposal arises from the indeterminate growth of plants. Due to the growth activity of the apical meristem, the cells that form the primary plant body are progressively *displaced* away from their point of origin at the growing tip toward the mature base of the root or stem. Primary growth at the cell level can be analyzed by kinematic methods that describe the movements of points or bodies through space (Erickson and Silk 1980). By measuring the relative elemental growth rates of cells in different zones, one can determine the turnover rates of cells at specific locations in the tip (Silk and Erickson 1979; Silk et al. 1986). In the maize root, for example, the entire population of cells in the transition zone is displaced into the zone of elongation roughly every 4.7 h (Wendy Silk, personal communication). The turnover rate would, of course, vary depending on the species and growth conditions, but the problem remains that the cells in this developmental zone are constantly being displaced. *The continuous displacement of cells out of the “brain-like command center” is incompatible with the formation of the stable processing networks capable of generating consciousness, feelings, and volition*. Nor is consciousness even necessary, because growing root tips process environmental stimuli by ordinary molecular signaling within and between their cells, by releasing hormones, Ca^2+^, and other known molecules to effect a response (Chaiwanon and Wang 2015; Taiz et al. 2015; Kong et al. 2018).

To be fair, it is important to point out that only *some* proponents of plant consciousness make the claim for a brain-like command center. Others, by contrast, accept its absence but argue that this absence does not preclude consciousness (Calvo 2017; Trewavas 2017). By analogy to the social insects, they advocate a distributed or “swarm intelligence,” stating that consciousness arises collectively from interactions between many tissues throughout the plant body. However, we question whether *swarm* intelligence has any relation to *individual* consciousness—and, there is considerable doubt whether “collective consciousness” even exists as an entity (Feinberg and Mallatt 2016a: p. 197; Friedman and Søvik 2019; Ginsburg and Jablonka 2021).

### Claim 7: plants show proactive, anticipatory behavior

Proponents claim that plants exhibit proactive behaviors, not just reactive ones, and that this intentional, proactive behavior indicates consciousness (Calvo 2017; Calvo and Friston 2017; Trewavas 2017; Latzel and Münzbergová 2018). Most of their examples involve the growth of roots, shoots, or climbing vines toward a goal or away from harm (e.g., Shemesh et al. 2010). But these examples always involve sensing and following a stimulus trail (“responses to stimuli”; “proactively sampling”: Calvo et al. 2017; Calvo and Friston 2017), which is reactive, not proactive. Rather than reflecting consciousness, it seems that plant growth patterns are preprogrammed to follow environmental clues. Truly proactive behavior that indicates consciousness would be to find the goal *in the absence of a sensory trail*, based on a mental map of the surrounding environment (Klein and Barron 2016; Feinberg and Mallatt 2018: p. 58) and on memories of this mapped space (Feinberg and Mallatt 2016a: pp. 114-115).

An example of true, planned, proactive behavior comes from experiments on spartaeine spiders (Tarsitano and Jackson 1997; also see Cross and Jackson 2016 and Perry and Chittka 2019). In the experiment, each spider started at the top of a tall cylinder where it could view two above-ground perches below it, on one of which was a prey. To get to the prey, the spider had to climb down from its cylinder onto the ground, from which the prey was no longer visible, and then choose between two paths made of bent poles, one of which led to the perch with the prey and the other to the perch without the prey. The spider walked along these poles, whose bends assured the spider had to go back and forth in “detours” and reach the perches indirectly—and the prey remained invisible until the spider climbed onto the perch. Even though they had never experienced the apparatus before, the spiders chose the correct route to the prey significantly more frequently (usually 2 to 4 times more) than they chose the wrong route. A key point is that there was no sensory trail to follow: the spider saw the prey only at the start, and the prey was imbedded in clear plastic so there was no olfactory cue to track. This means the spiders scanned and planned their routes in advance and formed some sort of mental representation of where to go. This is what we mean by proactive, conscious behavior. Plants have not been shown to meet the criteria for this behavior, because to date the experiments with plants have not removed access to the stimulus trail.

Instead plant movements resemble those of the roundworm *Caenorhabditis elegans* (*C. elegans*), which is the representative *non*conscious animal (Barron and Klein 2016: Klein and Barron 2016). When foraging in soil for its bacterial food, this worm continually uses many senses (taste, smell, touch, moisture) to track and find the richest bacterial patches (Ardiel and Rankin 2010; also see Gang and Hallem 2016), and it usually succeeds, especially when, upon losing the trail, it conducts a thorough and patterned search.

The preprogrammed searching of *C. elegans*, as a characteristic nonconscious behavior, resembles the winding growth movements of plants (circumnutation) that help them to find targets. It is therefore wrong to claim (Calvo 2017) that of these two organisms, only *C. elegans* lacks “goal-directed behavior” (incorrect because foraging is goal-directed by definition, the goal being to find the food or other resource). It is also erroneous to claim that “anything beyond their immediate surroundings eludes *C. elegans*,” because *C. elegans* follows the sensory trail out of its immediate surroundings and eventually finds distant food. And to claim that *C. elegans* “is unable to go beyond the here and now” is also incorrect because this worm *persists* in following the sensory trail for as long as it takes to achieve the goal. These observations show that certain nonconscious organisms can do impressive things without any proactive behavior. Another splendid example is the efficient foraging by fungal mycelial networks in the forest floor (Fricker et al. 2017), fungi also being nonconscious by our criteria. And recall how impressive but nonconscious foraging can be aided by attractant breakdown (Claim 1 above: Tweedy et al. 2020).

### Claim 8: plants show classical associative learning, which indicates consciousness

**Fig. 5:**
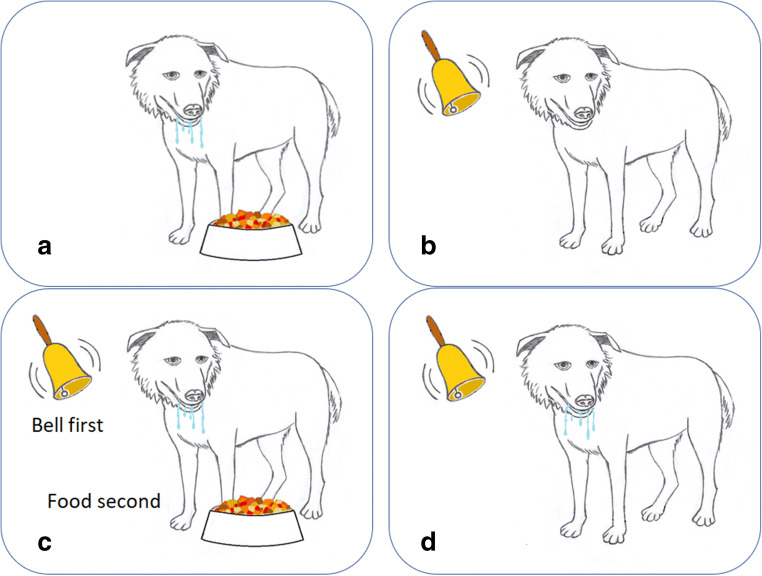
Classical associative learning, step by step. Here, a dog learns from a ringing bell (conditioned stimulus) that is presented prior to the smell of food (unconditioned stimulus) to salivate in response to the bell sound alone. Above, the pretraining steps show that the food alone induces drooling (**a**) but the bell alone does not (**b**). Training (**c**) rings the bell before presenting the food. After training (**d**), the bell alone induces drooling

To start with some background information, ethologists divide associative learning into two types. The first type is classical or Pavlovian learning (the simpler type). This is learning to associate a new stimulus with one that already causes an established behavior. An example is Pavlov’s dogs (Fig. 5). The second type is operant or instrumental learning (the more advanced type), which is learning from experience to change a behavior. For example, when a lab rat in a box accidentally pulls a lever, obtains a food reward, and learns to press the lever after a few trials, it exhibits operant learning.

Adelman (2018) recently reviewed the literature on whether plants have associative learning, with most of the studies having been done in the 1960s. His findings were a mixture of negative and positive results, but that none of the positive results had been replicated. Gagliano et al. (2016) claimed to have shown Pavlovian learning in growing pea seedlings, but Markel (2020a, b) could not replicate this, indicating problems with the controls in the original study.

A second study also reported associative learning in plants (Latzel and Münzbergová 2018: their Fig 3). In the main part of this study, rooting ramets of the clonal wild-strawberry *Fragaria vesca* were “trained” to grow onto nutrient-rich patches of soil either in the light (attractive) or the shade (less attractive), and then were monitored to see if they preferentially grew into lighted or shaded patches in the absence of nutrients. The results were a spotty mix of positives and negatives: plants that had been trained on lighted patches sent a significantly greater *biomass* of ramets to the lighted patches (*p* < 0.05) as predicted, but not a greater *number* of ramets; and then the *opposite* relationship between number and biomass inexplicably occurred for ramets that had been trained in the shade (except that the *p* values here were not quite significant—between 0.05 and 0.1). And another, “epigenetic,” part of the study used a chemical spray to demethylate the *Fragaria* plants’ genomes with the intent of erasing any ability to learn. There, however, the single statistically significant effect was “exactly the opposite” of that predicted. Yet all these negative and inexplicably opposite findings, both in the main study and the epigenetic experiment, were never addressed. Instead, the authors treated the results as though they were positive and significant.

We conclude that classical learning in plants remains unproven. But with regard to plant consciousness, *it does not matter either way because classical learning has always been considered nonconscious* (Goldman 2012; Rolls 2014; Rehman et al. 2020). Classical learning in the sense of behavioral adaptation to associations between two cues is fully explainable by changes of synaptic connectivity. This can occur without any complex perceptual or motor integration; e.g., at the simple level of reflex pathways like the gill-withdrawal reflex of the sea hare, *Aplysia californica* (Kandel and Schwartz 1982; Kandel 2009).

**Fig. 6:**
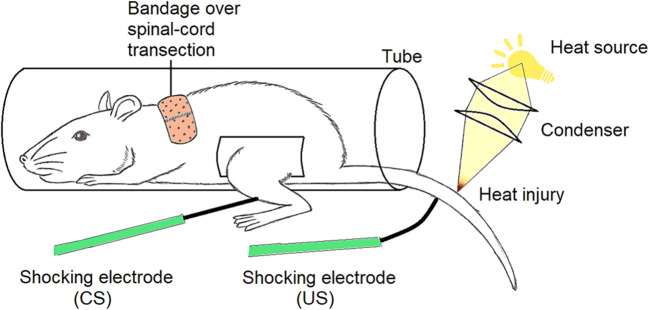
Classical learning by a spinal cord. Drawing is modified from Huie et al. (2015). The spinal cord was transected in the upper thorax 1 day before the learning experiment, so it was isolated from the brain. CS means conditioned stimulus (leg shock) and US means unconditioned stimulus (tail shock). During training, the mild shock to the leg is given just before an antinociceptive shock to the tail, the latter being a shock that naturally diminishes tail flick in response to the focused heat. With learning, the leg shock diminishes this tail flick when given alone (that is, it increases the latency time), having become antinociceptive

The clearest demonstration that classical learning is not conscious is that the isolated spinal cord of a human or rat can learn classically (note: we adopt the dominant view that the spinal cord is not conscious: Koch 2018). The relevant experiment is shown in Fig. 6 (Joynes and Grau 1996). Here, a rat’s spinal cord learns to associate a mild shock to the leg with an antinociceptive shock to the tail so the tail becomes less responsive to nociceptive heating. The interpretation is that, through classical learning, the leg shock has taken on a new, antinociceptive role**.**

**Fig. 7:**
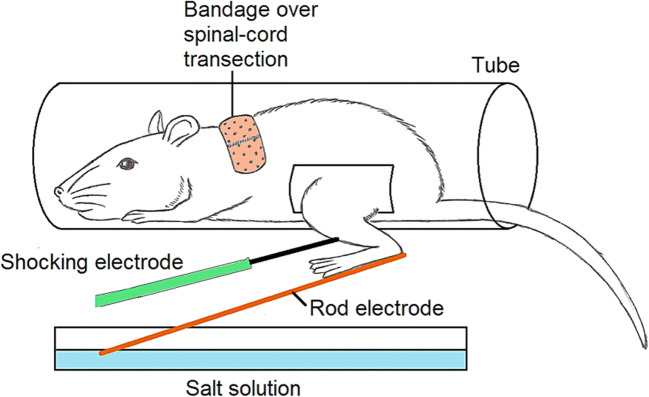
Limited operant learning by an isolated spinal cord. In the picture, the rod electrode also acts as a plate for the rat’s foot. A shock from the shocking electrode causes the leg to lift up, after which the leg naturally drifts down so the rod electrode enters the salt solution, completing a circuit that delivers another shock. As the cycle keeps repeating, the time it takes for the leg to drift down increases as the cord learns to delay the next punishing shock. Picture modified from Grau et al. (1998)

An isolated, nonconscious spinal cord can even learn *operantly*. The relevant experiment (Grau et al. 2006) trained a rat’s leg to lift up for a longer time, to avoid a punishing shock (Fig. 7). This is true operant learning, but it is limited to a single kind of response and has no learning flexibility. Still, even limited operant learning is beyond anything ever found in plants. Neither it nor classical learning, if at all present in plants, would mean they are conscious.

### Claim 9: plants communicate with each other in a purposeful manner and, hence, have conscious self-recognition

The exchange of volatile organic chemicals or other signals between plants has been interpreted as an adaptive behavior resembling cognition (Karban 2008; Leopold 2014; Trewavas 2016). Moreover, signaling between plants was taken as evidence that they distinguish between self and alien; i.e., for self-recognition (Trewavas 2017). Collective behavior of plant communities has, consequently, been interpreted as cooperative behavior indicating social cognition, intelligence, and thought (Karban 2008; Baluška and Manusco 2020). None of these observations requires consciousness, cognition, or collective planning. Exchange of signals between organisms is a widespread phenomenon in biology, beginning at the level of collective behavior in bacterial biofilms (Prindle et al. 2015). The communication occurs because all organisms evolve to detect (via receptors) every relevant and beneficial external stimulus, including molecules emitted by other organisms. And because all living organisms are defined by borders, they have an elementary distinction between self and alien. This distinction can be complex and adaptive, as, for example, in immune systems (Abbas et al. 2019). It does not, however, reflect or constitute consciousness.

Given these considerations, proponents can only argue that plant communication indicates consciousness if every living organism is conscious, including every bacterium—a highly problematic argument, as pointed out in Claim 1 above.

### Claim 10: detailed hypotheses, predictions, and models can substitute for hard evidence of plant consciousness

The proponents of plant consciousness in effect make this claim about the value of reasoned speculation. They do so by piling theory upon theory far beyond the evidence, as exemplified by this quote from Calvo et al. (2017: p. 2866). We italicized and underlined the many words and phrases that indicate uncertainty and speculation.

We have used some very old and modern literature to indicate unanswered questions about electrical signaling. The reticulated excitable phloem system described above *offers a potential* for assessment of signals and *perhaps* their prioritization. The bioelectric field in seedlings and in polar tissues *may also act* as a primary source of learning and memory. But *we suspect* that with time and experience, the developing phloem becomes increasingly cross-linked and memory *could* then reside in the electrical capabilities determined by numbers and characteristics of the cross linking. Local phenotypic changes to accommodate local environmental situations are characteristic of the behaviour of the self-organizing plant, and *maybe*, the bioelectric field coordinates with the electrical system to provide for the characteristics of self-organization. Both local and long distance changes are characteristics of higher plants. The vascular network is a complex interactive system, and once stimulated, it *has the potential* for assessment through *possible* feedback and alterations of connection strength. Animal-plant similarities being reported in the last decade point toward an electrochemical equivalency at the level of the nervous system elements (Baluška 2010), integrated by spatiotemporal dynamics (Masi et al. 2009). Whether it should be regarded as a functional equivalent to a fairly primitive brain *cannot be determined until its properties are more clearly defined by research*.

This article commenced by pointing out that lack of obvious movement in plants has led to incorrrect suppositions about a nervous control. With recognition that this highly branched excitable plant nervous system *might* act holistically, some issues that have dogged this area of research *might* be better understood.

On first consideration, it seems unfair to criticize such extreme speculation, because the authors explicitly stated that the goal of their article was “to indicate unanswered questions” about the topic of sentience. However, these are not one-time questions but ideas they believe to be true and continually promote in their publications without evidence (Baluška et al. 2009; Calvo 2017; Calvo and Trewavas 2020; Trewavas et al. 2020). Multiple speculative leaps without hard evidence are not only bound to introduce fatal errors in the chain of argument, but they also make the endeavor overly complex.

So far, we have examined ten claims for plant consciousness and none has held up.

## The alternate hypothesis of Feinberg and Mallatt

In order to analyze the final two claims for plant consciousness, we must first present a hypothesis by which to judge these claims. This hypothesis, called neurobiological naturalism, was developed by Todd E. Feinberg and one of us (JM) (Feinberg 2012; Feinberg and Mallatt 2013, 2016a, b, 2018, 2019, 2020). One of its goals was to identify which organisms have consciousness. To this end, we started with just two, logical assumptions.

### Assumption 1: affective (emotional) consciousness

From what assumption did we deduce which organisms have emotional consciousness? We assumed that emotions could be revealed by the capacity for operant learning from experience, because such reward- and punishment-induced learning goes with positive and negative emotions in humans. But we knew that *simple* operant learning can be *non*conscious (Fig. 7), so the criterion we chose is *high-capacity* operant learning: learning a brand-new behavior that uses one’s whole body (Feinberg and Mallatt 2016a: pp. 152-154). For example, a rat reveals emotional attraction when it has learned to walk to a lever and press the lever for a food reward. We adopted this assumption because it is *double* evidence of emotional feelings. That is, the existence of emotion is suggested by both (1) the initial attraction to a reward, and (2) recalling the learned reward to motivate behavior.

The only organisms that fit this criterion for affective consciousness are the vertebrates (all), arthropods (all), and cephalopods (octopus, squid, cuttlefish). Also see the similar theory of Bronfman et al. (2016) and Ginsburg and Jablonka (2019, 2021), with which we agree. Now we are ready to evaluate the claim that plants have affective consciousness.

### Claim 11: plants show affective (emotional) consciousness

Gagliano (2017) advocated affective consciousness in plants, by saying that their classical associative learning indicates “internal value systems based on feelings.” Her term “value systems” confirms that she was talking about the emotional feelings of *affective* consciousness, because “internal value” refers to *valence*, meaning the affective qualities of good = attractiveness and bad = averseness (Frijda 1986). We already refuted Gagliano’s claims for affects in plants, by showing that classical learning is nonconscious (see Claim 8 above).

Next we turn to the other type of primary consciousness, the image-based type.

### Assumption 2: image-based consciousness

For this type, we assumed that any organisms that demonstrably encode maps of the surrounding environment and of their bodies—from multiple senses such as vision, smell, touch, and hearing—will experience these mapped simulations consciously (Feinberg and Mallatt 2013, 2016a). It seems reasonable to assume that if a brain or body expends the energy to assemble such detailed maps, then it will use them, say, as mental reference images for moving and operating in the world.

**Fig. 8:**
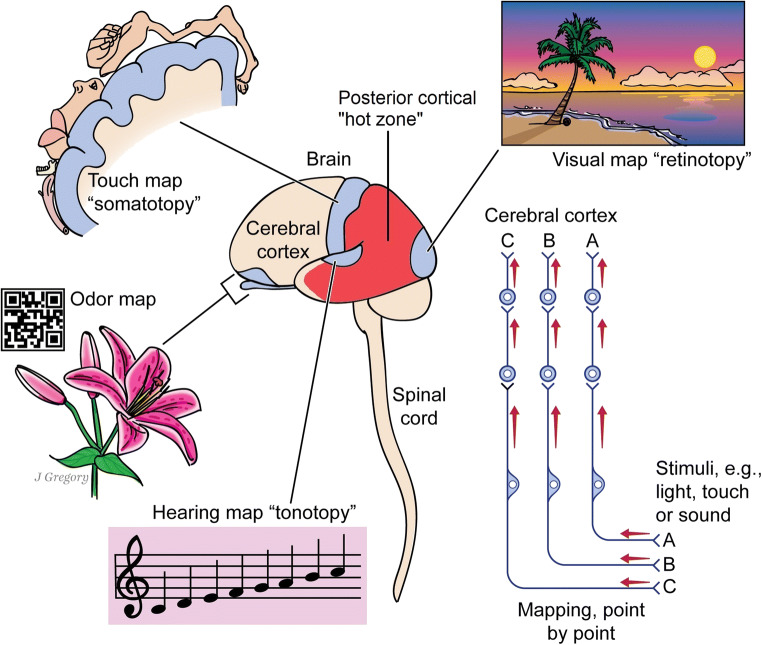
Neuronal sensory maps in the human nervous system. For each sense, a path of several neurons (far right) is a hierarchy that carries signals up to the brain, keeping a point-by-point mapping (A, B, or C) of the outside environment or a body structure. On reaching the cerebral cortex, this leads to the mapped neural representations that are shown around the brain. Information from the different senses is combined for *multisensory integration* (Stein et al. 2020), especially in the posterior cortical hot zone (Koch 2019: p. 61). Here, this seems to lead to a unified, all-sense map of the world that characterizes consciousness. This illustration is from Feinberg and Mallatt (2018). Used with permission from © Mount Sinai Health System

Figure 8 shows such mapped neuronal representations in humans up to the higher brain (cerebral cortex). Each of these pathways is known and has been documented (Brodal 2016). Other investigators have also related this kind of mapped representation to consciousness (Edelman 1989; Kaas 1997; Damasio 2010).

**Fig. 9:**
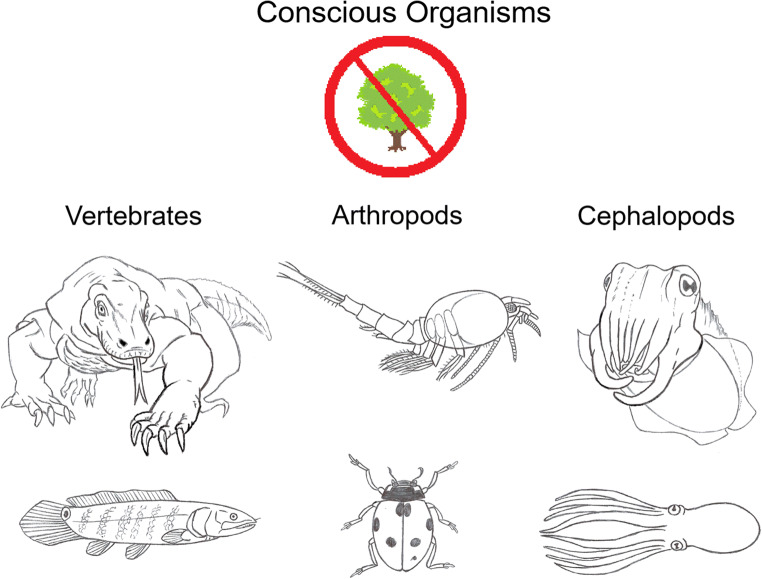
Summary figure showing that the conscious organisms, all of which we deduced to have both affective and image-based consciousness, do not include plants. The vertebrates are Komodo dragon *Varanus* and bowfin fish *Amia*. The arthropods are crustacean *Nebalia* and ladybug beetle *Coccinella*. The cephalopods are a cuttlefish and an octopus

This sensory mapping is only documented to exist in the nervous systems of certain animals, namely in all the vertebrates and arthropods, and in cephalopod molluscs (Feinberg and Mallatt 2016a). Therefore, these are the clades with image-based consciousness, and they are the *same* clades we found above to have *affective* consciousness (Fig. 9). They are also the animals with the most complex brains. Now we are ready to evaluate the claim that plants have image-based consciousness.

### Claim 12: plants have image-based consciousness, based on internal representations

A paper that was coauthored by the main proponents of plant consciousness claimed that within a bacterium “the environment is internally mapped” and that this likely holds for plants as well (Calvo et al. 2020). That paper also suggested that groups of cells within a plant’s body combine to construct an image, through “somatic mosaics.” But no evidence was presented for any of this. And the proponents effectively admit that too little is known about electrical signaling in the phloem vasculature to tell if phloem carries any mapped sensory information: see the quote from Calvo et al. (2017) in Claim 10 above.

## The evolution of consciousness

We have found that two separate lines of reasoning—one about affective consciousness and the other about image-based consciousness—agree that vertebrates, arthropods, and cephalopods are the only conscious organisms and that plants are not included. Consciousness must have appeared independently by convergent evolution in each of the three animal lines, because reconstructing their history indicates their last common ancestor lacked a brain (Northcutt 2012).

Note that the assumptions from which we deduced which organisms are conscious—the assumption of mapped representations for image-based consciousness, and of high-capacity operant learning for affective consciousness—have little to do *per se* with locomotor ability or fast mobility, yet they identified the most mobile clades of animals as the conscious ones (Fig. 9). Thus, our reasoning has independently reinforced the standard view that consciousness can only evolve in highly mobile organisms (Merker 2005; Taiz et al. 2019).

## Conclusions

This paper presents new arguments against plant consciousness, the most important of which are:

A. Plants do not show proactive behavior.
B. Classical learning does not indicate consciousness, so reports of such learning in plants are irrelevant.
C. The considerable differences between the electrical signals in plants and the animal nervous system speak against a functional equivalence. Unlike in animals, the action potentials of plants have many physiological roles that involve Ca^2+^ signaling and osmotic control; and plants’ variable potentials have properties that preclude any conscious perception of wounding as pain.
D. In plants, no evidence exists of reciprocal (recurrent) electrical signaling for integrating information, which is a prerequisite for consciousness.
E. Most proponents of plant consciousness also say that all cells are conscious, a speculative theory plagued with counterevidence.

Our 12 counterarguments are important to the future of plant biology, because dubious ideas about plant consciousness can harm this scientific discipline. We foresee three types of harm. First, not only does the notion of plant consciousness mislead the general public, but it also can generate mistaken ideas about the plant sciences in young, *aspiring plant biologists*. Second, the strong, romantic appeal of plant consciousness could influence public and private *funding agencies* to fund projects that are based on its fallacies. Third, public acceptance of plant consciousness could affect research *regulation*. For instance, could research on genetically modified plants face even more resistance if plants were regarded as conscious? How might laboratory-research regulations be impacted when scientists are seen to perform invasive manipulations on plants that feel pain?

These are not idle concerns. Articles that promote plant neurobiology thinking are increasingly finding their way into respectable scientific journals—even top-tier journals (Calvo and Friston 2017; Tang and Marshall 2018; Baluška and Manusco 2020; Calvo et al. 2020). This is most regrettable, and hopefully our article, by putting the record straight, will reverse this trend. In conclusion, we feel we must speak forcefully: plant neurobiologists have become serial speculationists. The ratio of speculation to data in their oeuvre is astronomically high. If they want to form a sensible hypothesis and then test it with real experiments, that is fine, but the prolific speculating and fantasizing need to stop.

## Data Availability

Not applicable.
